# Understanding Patterns of Care for Older Adults: Data to Action

**DOI:** 10.23889/ijpds.v9i5.2746

**Published:** 2024-09-18

**Authors:** Beliz Acan Osman, Fernando Maldonado, Suelen Goes, Nirmal Sidhu, Amanda Hutton

**Affiliations:** 1 Saskatchewan Health Quality Council

## Objective and Approach

Older adult population is growing rapidly, the current system is not adequately designed to meet the needs of individuals to live safely and independently in the place of their choice.

To understand the variations in care and identify where to intervene, we examined the transitions in care of community-dwelling older adults (COA) leading up to 24-hour assisted living at special care homes (SCH).

## Approach

We conducted a retrospective-cohort study using health administrative data from a Canadian province with a long history of comprehensive, longitudinal data. Using process mining techniques, health service utilization of COA, 65 and older, was mapped as a journey rather than independent care events. We examined the common patterns experienced leading up to SCH admission by sequencing transitions between care settings. Consideration was given to geography that the individual resides in and their health status to understand variations.

## Results

Overall health utilization patterns revealed predominant reliance on acute care and SCH admissions. However, geographic variations were identified (regions and urban/rural). More than 2000 transition patterns between emergency department, hospital, community, and SCH were observed.

## Conclusion

The ability for COA to age in place is dependent on the individual, where they live, and the availability of and access to services to meet their needs. To minimize undesirable and early admissions to SCH, a multifaceted, collaborative approach to align community-based supports for COA is required. The results of these analyses will help inform areas in which to focus interventions and serve as a baseline while evaluating impact.

